# IgCAMs redundantly control axon navigation in *Caenorhabditis elegans*

**DOI:** 10.1186/1749-8104-4-13

**Published:** 2009-04-02

**Authors:** Valentin Schwarz, Jie Pan, Susanne Voltmer-Irsch, Harald Hutter

**Affiliations:** 1 Max Planck Institute for Medical Research, Jahnstr., 69120 Heidelberg, Germany; 2 Simon Fraser University, University Drive, Burnaby, BC, V5A 1S6, Canada

## Abstract

**Background:**

Cell adhesion molecules of the immunoglobulin superfamily (IgCAMs) form one of the largest and most diverse families of adhesion molecules and receptors in the nervous system. Many members of this family mediate contact and communication among neurons during development. The *Caenorhabditis elegans *genome contains a comparatively small number of IgCAMs, most of which are evolutionarily conserved and found across all animal phyla. Only some of these have been functionally characterized so far.

**Results:**

We systematically analyzed previously uncharacterized IgCAMs in *C. elegans*. Green fluorescent protein reporter constructs of 12 IgCAMs revealed that expression generally is not confined to a single tissue and that all tissues express at least one of the IgCAMs. Most IgCAMs were expressed in neurons. Within the nervous system significant overlap in expression was found in central components of the motor circuit, in particular the command interneurons, ventral cord motoneurons as well as motoneurons innervating head muscles. Sensory neurons are underrepresented among the cells expressing these IgCAMs. We isolated mutations for eight of the genes showing neuronal expression. Phenotypic analysis of single mutants revealed limited neuronal defects, in particular axon navigation defects in some of the mutants. Systematic genetic interaction studies uncovered two cases of functional overlap among three and four genes, respectively. A strain combining mutations in all eight genes is viable and shows no additional defects in the neurons that were analyzed, suggesting that genetic interactions among those genes are limited.

**Conclusion:**

Genetic interactions involving multiple IgCAMs affecting axon outgrowth demonstrate functional overlap among IgCAMs during nervous system development.

## Background

The brain is a rather complex organ. Its building blocks, the neurons, migrate to the proper places, differentiate into the correct subtype and send out neuronal processes that eventually connect to the appropriate target cells to form neuronal circuits. Many of these events require cell communication and differential cell adhesion. The most prominent families of adhesion molecules mediating contact and communication among neurons are cadherins, integrins and members of the immunoglobulin superfamily (IgCAMs). IgCAMs form the largest and most diverse family of adhesion molecules in the nervous system. Immunoglobulin domains are found in many different proteins, typically in multiple copies and in combination with a number of other extracellular domains, most notably fibronectin III (FnIII) domains. Well-characterized members of this family are the neural cell adhesion molecule NCAM and members of the L1 subfamily (for a recent review see [1]). NCAM mutant mice show various mild neuronal defects, such as a reduced olfactory bulb [2] and defects in the maintenance of mossy-fiber projections in the hippocampus [3]. Mutations in L1 lead to a variety of developmental defects in the nervous system, including pathfinding errors of axons in different parts of the brain [4-6]. In humans mutations in L1 lead to mental retardation, hydrocephalus, agenesis of the corpus callosum and optic nerve atrophy [7]. IgCAMs have been shown to mediate homophilic and heterophilic adhesion and numerous interactions among various family members have been documented [8]. Furthermore, members of this family act as receptors for secreted axon guidance signals, indicating that the label 'cell adhesion molecule' captures only part of their function [9]. There is extensive cross-talk between IgCAMs and heterologous receptor systems. The list of interacting partners includes receptor tyrosine kinases, integrins, neuropilins and cadherins [1,10]. In other words, IgCAMs comprise a diverse and large family of adhesion/receptor molecules with a variety of different functions during nervous system development.

**Figure 1:**
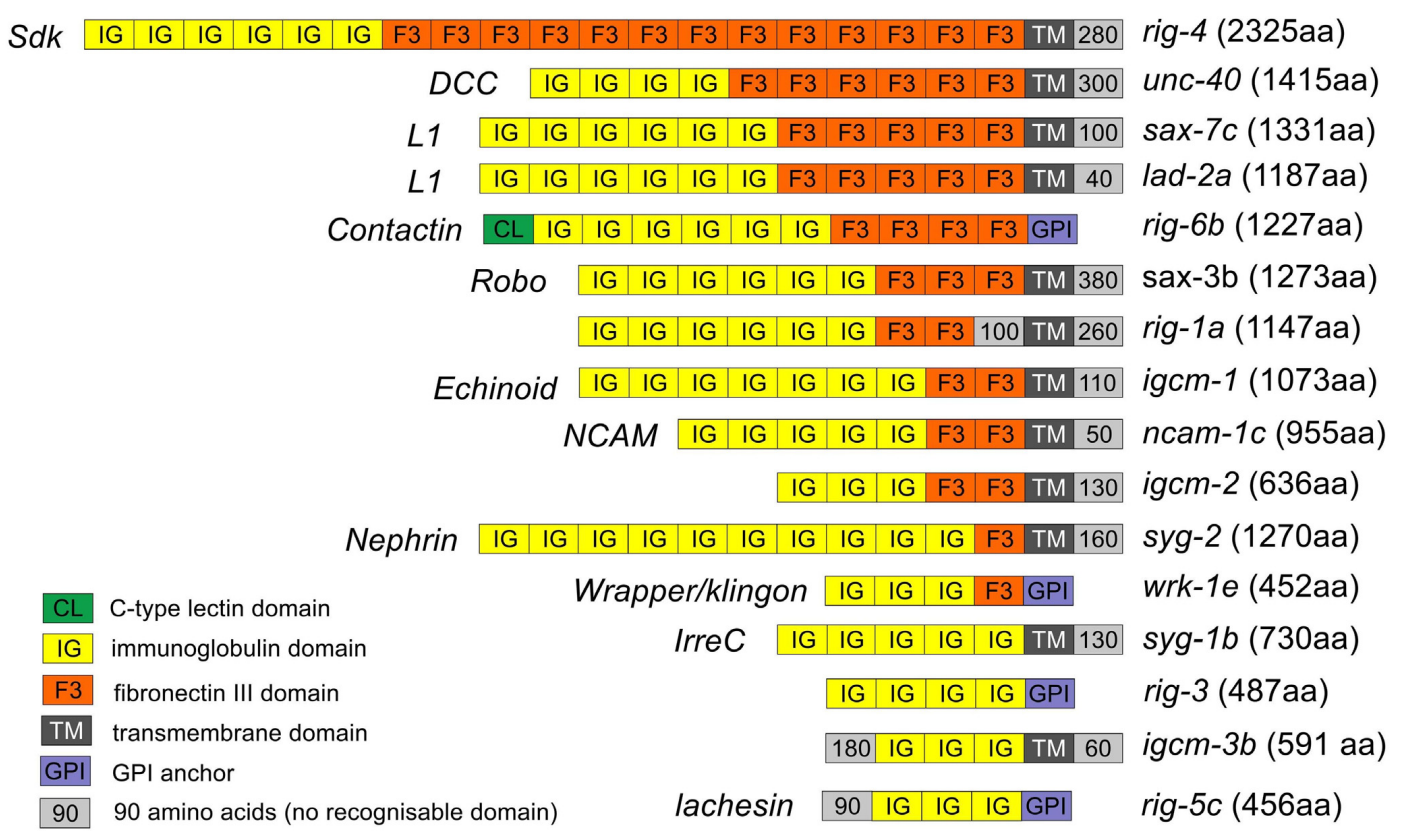
**Domain organization of IgCAMs in *C. elegans***. Protein domains are depicted as boxes. The size of the box does not reflect the size of the domain. *C. elegans *gene names are listed on the right side; numbers in brackets behind the names correspond to the protein size in amino acids (aa). Names of *Drosophila *or vertebrate orthologs are given on the left side.

The precise definition of what constitutes an IgCAM is not trivial given the large range of proteins containing immunoglobulin domains and the observation that several members of the family are better defined as receptors rather than adhesion molecules. We follow here the core of the original definition based on structural features of the proteins [11]. IgCAMs defined in this way contain one or more immunoglobulin domains and are involved in cell recognition events. For the purpose of this study we restrict this and define IgCAMs as having at least three immunoglobulin domains and no additional domains other than FnIII domains in the extracellular part. This definition excludes smaller Ig-containing proteins [12] as well as proteins like UNC-5, which contains thrombospondin type 1 domains [13]. Following this definition, the *Caenorhabditis elegans *genome contains 17 IgCAMs [14,15]. Twelve of the IgCAMs have identifiable homologues in other animals, and the remaining five potentially arose within the nematode lineage. Subfamilies within the IgCAMs are mainly defined by their domain composition; for example, the L1 subfamily is characterized by six Ig domains followed by five FnIII domains. The domain composition in all subfamilies is almost completely preserved in the *C. elegans *homologues, a striking evolutionary conservation given that Ig and FnIII domains are among the most 'promiscuous' protein domains, which have at the same time been used to generate a wide variety of different proteins. With the exception of the L1 subfamily, which has two members in *C. elegans *(Figure 1), subfamilies are represented by only one member, that is, there is one NCAM homolog, one member of the contactin subfamily and so on.

Seven of the 17 *C. elegans *IgCAMs have been functionally characterized. Among the first to be identified were receptors for secreted axon guidance cues: UNC-40 as receptor for UNC-6/netrin [13,16] and SAX-3/Robo as receptor for slit [17]. The L1 homologue LAD-1/SAX-7 acts as a homophilic adhesion molecule to hold neurons in place and is also required for morphogenesis [18-21]. SYG-1 together with SYG-2 controls placement of the synapses of the HSN neurons [22,23]. More recently, WRK-1 has been shown to be involved in axon navigation at the ventral midline [24] and a second member of the L1 subfamily, LAD-2, functions as a co-receptor for Semaphorin-mediated axon guidance [25]. In summary, almost all IgCAMs characterized so far in *C. elegans *are involved in some aspect of neuronal development, most notably axonal navigation or synapse formation. This fact together with information about homologues in other animals makes IgCAMs the most obvious candidate family to search for additional neuronal adhesion molecules and receptors.

This study presents the characterization of a large number of previously uncharacterized IgCAMs in *C. elegans*. We began by analyzing their expression patterns and found that most had a neuronal expression component. We then isolated or obtained deletions in eight IgCAMs that show predominant neuronal expression. Phenotypic characterization revealed limited neuronal defects, predominantly axon guidance defects, in some of these single mutants. Various combinations of multiple mutants were generated to test for functional redundancy among those genes. This revealed an interaction among four IgCAMs affecting interneuron axon navigation in the ventral cord and three different IgCAMs being responsible for the direction of outgrowth of certain motoneuron commissures.

## Results

### Expression analysis

**Figure 2:**
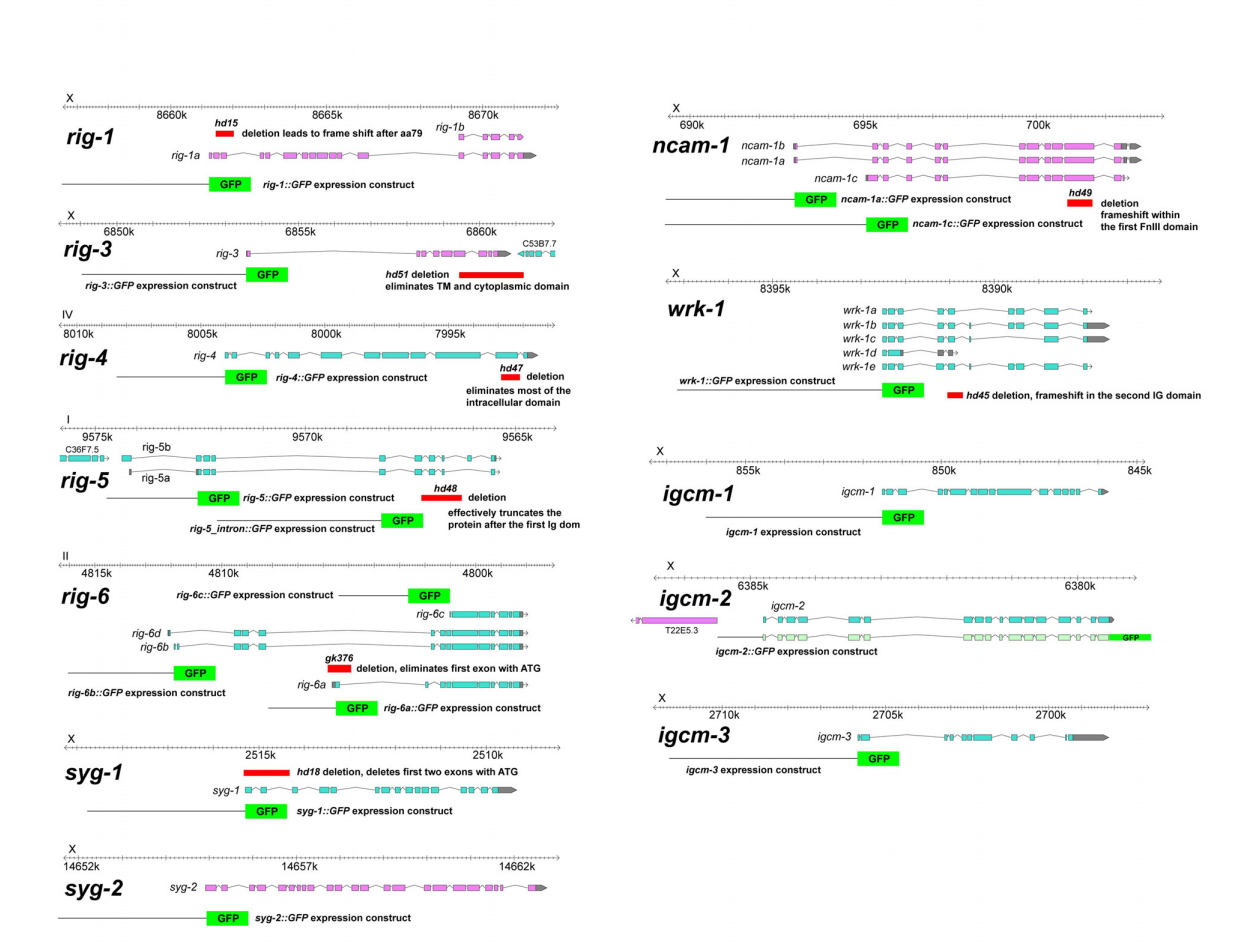
**Expression constructs and deletion alleles**. The genomic regions of the IgCAM genes studied here with the location of the mutations and promoter regions used for expression studies indicated.

To study the expression patterns of previously uncharacterized IgCAMs (Figure 1), we generated transgenic lines carrying green fluorescent protein (GFP) reporter constructs with the putative promoter regions of the genes. We tested for the presence of regulatory elements in large introns near the 5' end of the gene with additional reporter constructs, although this was not systematically done for every gene (Figure 2). Most IgCAMs are expressed in more than one tissue and the majority has a neuronal expression component (Tables 1 and 2). The only IgCAM with expression limited to a single tissue was *rig-3*, which is only expressed in neurons and some associated glia. All the major tissues (epidermis, muscle, digestive system, nervous system) expressed at least two different IgCAMs. GFP expression was typically first detectable in embryos towards the end of gastrulation. Expression typically began in only a few cells, with more and more cells expressing GFP during elongation phase. Cell identification in the embryo is difficult, but the overall pattern suggested that embryos up to the twofold stage generally show only part of the expression seen postembryoncially. Expression continued throughout larval development into adulthood.

**Table 1:** Tissue specificity in IgCAM expression

Gene	Epidermis	Muscle	Gastro-intestinal system	Reproductive system	Nervous system	Other
*syg-1*		Head muscle, vulva muscle			See Table 2	
*syg-2*	Vulva epithelium	Body wall muscle, vulva muscle			See Table 2	
*wrk-1*			Gut (embryo, early larval stages)	Distal tip cell	See Table 2	Coelomocytes, arcade cells
*ncam-1a/b*			Gut (embryo)		See Table 2	GLR cells
*ncam-1c*			Pharynx		See Table 2	GLR cells
*rig-1*	Hypodermis		Pharyngeal muscle		See Table 2	Amphid socket
*rig-3*	-	-	-	-	See Table 2	Amphid sheath cells
*rig-4*	Seam cells, vulva epithelium (weak)		Gut, pharyngeal muscle	Uterine valve cells	See Table 2	Excretory cell
*rig-5a/b*	-	-	Gut	-	See Table 2	-
*rig-5 intron*	-	-	g1 glands	-	See Table 2	-
*rig-6a*	Rectal epithelium	Body wall muscle, vulva muscle		Spermetheka	See Table 2	Excretory cell
*igcm-1*	Seam cells		Pharyngeal marginal and muscle cells		Labial sensory, CAN, phasmid sheath, a few head neurons	Arcade cells
*igcm-2*	Hypodermis (weak)		Gut	Gonad	A few head neurons	
*igcm-3*	Major hypodermis, vulva and rectal epithelium, seam cells		Pharyngeal muscle	Spermatheka		Arcade cells

**Table 2:** Neuronal expression of IgCAMs

Gene	Sensory neurons	Command interneurons	Interneurons: other	Motoneurons: head/tail	Motoneurons: ventral cord	Pharyngeal neurons
*syg-1*	ADF, ADL	AVH?	AIN, RIG, RIS, SAA?, SIA, SIB; RIF or SAB	RIM	DA, DB, DD, VA, VB	M3?, M4
*syg-2*			ALN, PLN, AIZ?, RIC?, PVT	RIM?	DA, DB, DD (in embryo only)	
*wrk-1*	ASI, ASJ, ASK, AWB, PLM?	AVA, AVE	AIB, AIN?, AIY, AUA, RIC, RIG, DVA?	RMD, RME, SMD, URA?, PDA	DA, DB, DD	
*ncam-1*	ASI, ASJ	AVB, AVE, PVC	AIB, AIN		DA, DB	M2, NSM
*rig-1*		AVA, AVB, AVD, AVE, PVC	AIB, AIN, AUA, AVH?, AVJ, RIC,	RMD, SMD		I2
*rig-3*		AVA				I1, I4, M4, NSM
*rig-4*			AIA?, RIA?, URX, RIH?	RME	DA (weak)	
*rig-5a/b*				RMD		
*rig-5intron*		AVD	AIN?, AUA?, RIC?, RIF?	RMD, SMDV		I2, MC, M4, M3?, M2?
*rig-6*		AVA, AVB, AVE, PVC	AIB, AUA, AVG, RIB, RIC, SAA, SIA, SIB, RIF	RIM, RMD, RME, SMD	DA, DB, VA, VB	M5, NSM, MC, I3, M1?

**Figure 3:**
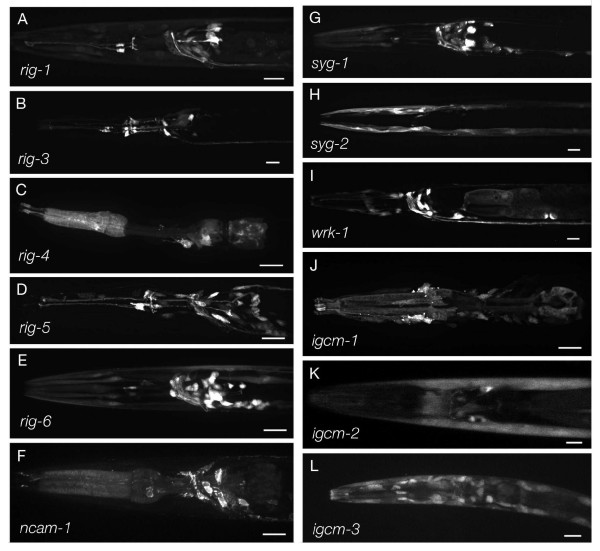
**IgCAM expression**. Representative pictures showing expression of strains carrying expression reporter constructs for **(A) ***rig-1*, **(B) ***rig-3*, **(C) ***rig-4*, **(D) ***rig-5*, **(E) ***rig-6*, **(F) ***ncam-1*, **(G) ***syg-1*, **(H) ***syg-2*, **(I) ***wrk-1*, **(J) ***igcm-1*, **(K) ***igcm-2*, and **(L) ***igcm-3*. All pictures show head regions of adult animals. Anterior is to the left. Scale bar: 10 μm.

Neuronal expression was studied in more detail (Table 2, Figure 3). The *C. elegans *nervous system of the hermaphrodite excluding pharyngeal neurons consists of 280 neurons in 103 distinguishable classes [26]. Of these, 56 expressed at least one of the IgCAMs studied here. Five of the IgCAMs are expressed in up to five different classes of pharyngeal neurons, leaving only four out of 20 pharyngeal neurons without IgCAM expression. Prominent sites of IgCAM expression are head ganglia with interneurons and motoneurons innervating head muscles as well as the ventral cord with motoneurons innervating body wall muscle (Additional file 1). The majority of the sensory neurons and most neurons located in the tail ganglia do not express the IgCAMs studied here. Some neurons express up to five IgCAMs, providing a basis for combinatorial action. Among those are RMD and SMD, two classes of neurons receiving extensive synaptic input from many other neurons that comprise the two major 'hubs' in the motor circuit innervating head muscles [26]. Other classes of neurons expressing a large number of different IgCAMs are all embryonic ventral cord motoneurons (DA, DB, DD) and some postembryonic ones (VA, VB). A final centre of IgCAM expression comprises the command interneurons innervating ventral cord motoneurons (AVA, AVB, AVD, AVE, PVC), almost all of which express three different IgCAMs. Apart from these 'hubs of expression', there is no clear overall correlation between the number of synaptic partners and number of IgCAMs expressed in a given neuron. In summary, IgCAM expression is concentrated on the output side of the nervous system (motor circuit), whereas the input side (sensory neurons) and primary interneurons connected to sensory neurons are underrepresented.

### Functional analysis: single mutants

For an initial functional analysis we decided to concentrate on eight genes with expression in neurons of the ventral cord motor circuit, since these seem to be a major centre of IgCAM expression. We isolated deletion alleles of *rig-1*, -*3*, -*4*, -*5*, *ncam-1*, *syg-1 *and *wrk-1 *(Figure 2; Additional file 2). A deletion in *rig-6 *was available through the *C. elegans *Gene Knock-Out Consortium. These deletions should substantially compromise or even completely eliminate gene function, since they result in frame-shifts and early truncations of the protein (Figure 2; Additional file 3).

Animals carrying mutations in any of these genes are viable, grow up to adulthood and reproduce without any obvious defects, indicating that none of these genes is essential for survival under laboratory conditions. The structure of the nervous system was examined with a number of GFP markers labeling either the entire nervous system or selected subsets of neurons. None of the markers used was misexpressed or revealed any neuronal migration defects, indicating that cell fate specification and cell migration in the nervous system generally are not affected in these mutants. Evaluating longitudinal axon tracts and cell body position of neurons with a pan-neuronal marker, we found significant defects in only two of the eight mutants tested. In both cases (*wrk-1 *and *rig-1*) axons aberrantly crossed the ventral midline switching between left and right ventral cord axon tracts. In addition, *wrk-1 *mutants were characterized by misplaced motorneuron cell bodies, which were occasionally found lateral to the ventral cord rather than between the left and right ventral cord axon tracts. To further characterize the defects in the ventral cord, we used a number of cell type specific markers. Motoneuron markers revealed motoneuron placement defects for DD/VD motoneurons but not for DA/DB motoneurons in *wrk-1 *mutants (Table 3). This confirms the observation with the pan-neuronal marker and suggests that these defects are limited to certain classes of motoneurons. In addition, we found minor defects in motoneuron commissure guidance towards the dorsal cord in *rig-6 *mutants and minor defects in the direction of outgrowth (left versus right) of the DA2/DB3 commissures in *syg-1 *mutants (Table 3). No other significant defects in motoneurons were found in any of the other single mutants. Three different markers were used to visualize pioneers (AVG, PVP) and follower interneurons (PVQ, command interneurons) in the ventral cord. Statistically significant defects were found only in *wrk-1 *mutants, which show ventral cord cross-over defects of PVP, PVQ and command interneuron axons, as already observed in an earlier study [24].

**Table 3:** Motoneuron defects in IgCAM mutants

	DD/VD motoneurons				DA/DB motoneurons				
		
Gene	Cell body	VC	Side	Navig.	Cell body	VC	Side	DA2/DB3	Navig.
**Wild type**									
*hdIs22*	1	4	12	7	0	0	3	0	1
									
**Single mutants**									
*rig-1(hd15) X*	4	4	12	9	0	3	1	0	3
*rig-3(hd51) X*	0	3	9	5	0	0	0	0	0
*rig-4(hd47) IV*	0	1	21	2	0	0	5	0	0
*rig-5(hd48) I*	0	2	6	15	0	1	0	0	0
*rig-6(gk376) II*	2	2	9	6	0	0	6	0	0
*rig-6(ok1589) II*	1	8	21	28*	0	1	6	0	0
*syg-1(hd18) X*	5	4	12	4	1	1	3	10*	1
*wrk-1(hd45) X*	27*	5	9	8	3	0	5	0	0
*ncam-1(hd49) X*	0	2	15	9	0	0	2	0	0
									
**Multiple mutants**									
*wrk-1 ncam-1 X*	28*	14^†^	17	15	1	0	8	0	0
*syg-1 wrk-1 X*	26*	11	29*	19^†^	0	0	7	13*	0
*syg-1 ncam-1 X*	1	7	23^†^	5	0	0	5	12*	0
***syg-1 wrk-1 ncam-1****X*	34*	4	18	12	3	0	6	18*	0
*rig-6 II; rig-4 IV; syg-1 X*	1	4	18	3	0	0	2	7*	0
*rig-6 II; rig-4 IV; wrk-1 X*	21*	3	15	6	3	0	12^†^	2	0
*rig-4 IV; syg-1 wrk-1 X*	24*	6	8	4	2	1	6	12*	0
*rig-6 II; syg-1 wrk-1 X*	19*	9	10	3	1	1	5	9*	0
*rig-6 II; rig-4 IV; syg-1 wrk-1 X*	35*	7	21	8	4^†^	4^†^	5	14*	0
*rig-6 II; rig-4 IV; ****syg-1 wrk-1 ncam-1****X*	22*	7	24^†^	4	2	0	4	22*	0
*rig-5 I; rig-6 II; rig-1 rig-3 X*	0	1	13	8	0	6^†^	7	0	1
*rig-5 I; rig-6 II; rig-4 IV; rig-1 ****wrk-1*** *rig-3 ****syg-1 ncam-1****X*	29*	13^†^	43*	20*	1	2	9	21*	0

Alleles used were: *syg-1 (hd18); wrk-1 (hd45); ncam-1 (hd49); rig-1 (hd15); rig-3 (hd51); rig-4 (hd47); rig-5(hd48); rig-6(gk376)*. Cell body: cell body outside ventral cord. VC: ventral cord crossover/outgrowth defects. Side: commissure growing on wrong side (DA2/DB3 excluded). DA2/DB3: commissures of those two motoneurons growing on wrong side. Navig.: commissure not reaching dorsal cord. n = 100, numbers in table are percent of animals with defects. *Significant with *p *< 0.01; ^†^significant with *p *< 0.05; χ^2 ^test.

### Functional analysis: multiple mutants

There are several possible explanations for the limited defects found in individual IgCAM mutants. Some of these genes could be involved in aspects of nervous system development not analyzed here. A second possibility is functional redundancy among the various members of this family, which have a rather similar domain organization and overlapping expression patterns. This can be tested by studying double (or multiple) mutants of genes suspected to act redundantly. We decided to address this question systematically for the entire set of eight genes for which we had mutations available. To keep the number of multiple mutant combinations manageable, we divided the genes into two groups based on their expression patterns. One group with expression predominantly in motoneurons consisted of *rig-4*, *rig-6*, *syg-1*, *wrk-1 *and *ncam-1*. The other group consisted of *rig-1*, -*3*, -*5 *and -*6*, genes with expression in the command interneurons. We systematically made double, triple, and quadruple (or quintuple in the case of the motoneuron subset) mutant combinations. All mutant combinations generated were viable and healthy, indicating that also none of the mutant combinations tested is essential for survival under laboratory conditions. No novel axon guidance phenotypes were observed with the markers used (Tables 3 and 4). All defects found in the single mutants were found in all multiple mutant combinations containing the single mutant in question, that is, we never observed suppression of any phenotype. For two of the phenotypes we observed enhancement of the defects in certain mutant combinations. In a *rig-1*, -*3*, -*5*, -*6 *quadruple mutant we found increased cross-over defects of command interneurons in the ventral cord with a penetrance of 30%. Defects in the strongest single mutant (14% in *rig-1*) are significantly milder and the other three single mutants show no significant defects (Table 4). In contrast to the quadruple mutant none of the triple mutant combinations shows any enhanced defects, suggesting synergistic effects among all four of the IgCAMs tested. While these four genes are all expressed in some of the interneurons showing defects (Additional file 1), no single interneuron expresses all four of them, implying some cell non-autonomous effects. Only one other defect was enhanced in any of the mutant combinations tested: directional outgrowth defects in DA2/DB3 commissures (left versus right) were increased to about 20% (from 10% in *syg-1 *single mutants) in all mutant combinations containing *syg-1*, *wrk-1 *and *ncam-1 *(Table 3). These genes are co-expressed in all DA and DB motoneurons, but curiously the defect was always limited to DA2 and DB3, indicating a strong positional effect, which is not reflected in the expression.

**Table 4:** Pioneer and interneuron defects in IgCAM mutants

Gene	VC entry	Inter-neuron	AVG	PVPL	PVQR	PVPR	PVQL
**Wild type**							
*rhIs4 hdIs26*	1	6	0	0	0	7	9
							
**Single mutants**							
*rig-1(hd15) X*	2	14	0	0	0	4	4
*rig-3(hd51) X*	0	4	0	0	0	9	9
*rig-4(hd47) IV*	0	0	0	0	0	7	7
*rig-5(hd48) I*	0	4	0	0	1	9	9
*rig-6(gk376) II*	1	6	0	0	0	7	7
*rig-6(ok1589) II*	0	1	0	0	0	5	5
*syg-1(hd18) X*	1	6	0	0	0	6	6
*wrk-1(hd45) X*	1	16^†^	0	0	0	34*	36*
*ncam-1(hd49) X*	1	5	0	1	1	12	12
							
**Multiple mutants**							
*rig-6 II; rig-4 IV; syg-1 X*	0	4	0	1	1	11	10
*rig-4 IV; syg-1 wrk-1 X*	2	9	0	4^†^	4^†^	35*	35*
*rig-6 II; syg-1 wrk-1 X*	6	11	0	3	3	40*	40*
*rig-6 II; rig-4 IV; syg-1 wrk-1 X*	1	18^†^	0	1	3	34*	35*
*rig-5 I; rig-6 II; rig-1 X*	5	12	0	0	1	8	8
*rig-5 I; rig-6 II; rig-3 X*	2	6	0	0	0	4	4
*rig-5 I; rig-1 rig-3 X*	7^†^	10	0	0	0	7	7
*rig-6; rig-1 rig-3 X*	4	8	0	0	0	6	6
*rig-5 I; rig-6 II; rig-1 rig-3 X*	4	30*	1	1	1	16^†^	14
*rig-5 I; rig-6 II; rig-4 IV; rig-1 wrk-1 rig-3 syg-1 ncam-1 X*	6	37*	0	0	2	48*	48*

Alleles used were: *syg-1 (hd18); wrk-1 (hd45); ncam-1 (hd49); rig-1 (hd15); rig-3 (hd51); rig-4 (hd47); rig-5(hd48); rig-6(gk376)*. All defects are ventral cord cross-over defects. VC entry: failure of axons to cross to right side upon ventral cord entry (rhIs4). Interneuron: *glr-1::GFP *expressing interneurons. n = 100, numbers in table are percent of animals with defects. *Significant with *p *< 0.01; ^†^significant with *p *< 0.05; χ^2 ^test.

To address the possibility that the subdivision of our mutants in two sets prevented us from detecting additional interactions, we combined all eight mutations into one strain. Even this strain (*rig-5 I; rig-6 II; rig-4 IV; rig-1 wrk-1 rig-3 syg-1 ncam-1 X *octuple mutant) is viable and grows well under laboratory conditions. Virtually every tissue expresses at least one of these IgCAMs and we examined all tissues in this mutant combination using Nomarski optics and polarized light microscopy. We found overall body morphology, pharyngeal structure, and appearance of the gut, gonad, body wall muscle, vulva muscle as well as the vulva itself to be normal (data not shown). We detected a low penetrance of distal tip cell migration defects apparent as extra turns of the gonad on the dorsal side in about 23% of the animals (Table 5) and a fairly penetrant egg-laying defect, with animals retaining more than the normal number of eggs in the body (Table 5). To determine which of the eight genes contribute to these phenotypes, we examined two quintuple and one quadruple mutant (Table 5). For the distal tip cell migration phenotype we found that in all these mutant combinations the penetrance of the phenotype dropped significantly, an indication that either all eight genes contribute to the phenotype or that this low penetrance defect is an unspecific side-effect of combining a large number of mutations in a single strain. For the egg-laying defect we observed that each mutant combination had defects with a penetrance similar to the octuple mutant, suggesting that there is little specificity in this phenotype as well.

**Table 5:** Non-neuronal defects in multiple IgCAM mutants

Gene	Egl	DTC migration
Wild type	5	1
*rig-5 I; rig-6 II; rig-4 IV; rig-1 wrk-1 rig-3 syg-1 ncam-1 X*	62*	23*
*rig-5 I; rig-6 II; rig-1 rig-3 X*	74*	8^†^
*rig-6 II; rig-4 IV; wrk-1 syg-1 ncam-1 X*	49*	8^†^
*rig-1 wrk-1 rig-3 syg-1 ncam-1 X*	73*	1

Alleles used were: *syg-1 (hd18); wrk-1 (hd45); ncam-1 (hd49); rig-1 (hd15); rig-3 (hd51); rig-4 (hd47); rig-5(hd48); rig-6(gk376)*. Egl: adult animals accumulating eggs in the uterus. DTC migration: animals with one gonad arm showing extra turns on the dorsal side. n = 100, numbers in table are percent of animals with defects. *Significant with *p *< 0.01; ^†^significant with *p *< 0.05; χ^2 ^test.

We used a pan-neuronal marker to evaluate several neuroblast migrations and the overall appearance of the nervous system. We found no gross nervous system abnormalities apart from the ventral cord defects already described and no defects in the migration of the Q neuroblasts (data not shown). In 20% of the animals one of the HSN neurons was not detected near the vulva, indicating a potential HSN migration defect. HSN neurons are required for egg-laying, and the migration defect might explain the egg-laying defects in some animals.

**Figure 4:**
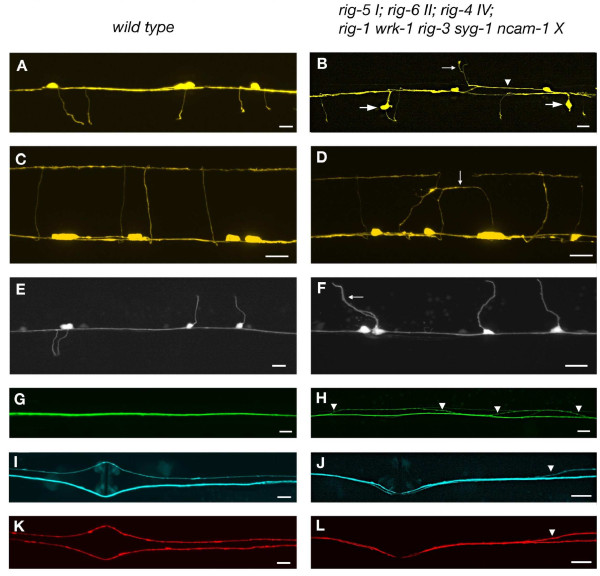
**Neuronal defects in IgCAM mutants**. Wild type is on the left and defects in *rig-5 I; rig-6 II; rig-4 IV; rig-1 wrk-1 rig-3 syg-1 ncam-1 X *mutant animals are shown on the right. Defects are indistinguishable from the defects seen in the single mutants and, therefore, representative of single mutants as well. Anterior is to the left. Scale bar: 10 μm. **(A-D) **DD/VD motoneurons (A-B ventral view, C-D lateral view). **(E, F) **DA/DB motoneurons, ventral view. **(G, H) ***glr-1::GFP *expressing interneuron axons. **(I, J) **PVP/AVG. **(K, L) **PVQ axons, same animal as in (I-J). Defects consist of axons extending in the left ventral cord tract (arrowhead in (B)), commissures growing on the left rather than the right side (small arrow in (B)), misplaced motoneuron cell bodies located outside the ventral cord region (large arrows in (B)), commissures not reaching the dorsal cord (small arrow in (D)), DA2/DB3 commissures growing on the left rather than the right side (arrow in (F)), interneuron axons crossing between left and right axon tracts (arrowheads in (H)), and PVPR and PVQL axons crossing into the right axon tract (arrowheads in (I, L)).

With respect to axon guidance defects in the motorcircuit, we did not see a further enhancement of any of the defects discussed so far (Tables 3 and 4 and Figure 4). This indicates that the genetic interactions discussed above are specific to the set of genes tested and that we probably did not miss any further genetic interactions among the genes with respect to the phenotypes analyzed.

## Discussion

### Evolutionary conservation of the IgCAM family

IgCAMs are found in every animal phylum and the defining protein domain, the immunoglobulin domain, has been used to create a wide variety of proteins with different functions both inside and outside the cell. The *C. elegans *genome contains 78 genes with immunoglobulin domains [14,15,27]. Of these, 17 constitute the core group of proteins commonly referred to as IgCAMs, defined here as cell surface proteins with at least three Ig domains and possibly FnIII domains. Eight more genes encoding putative secreted proteins with two Ig domains ('zig' genes) as well as four single Ig containing proteins ('oig' genes) are not considered here. Characterized members of this group are important for maintenance of axon position in the ventral cord [12]. Most subfamilies of IgCAMs known in vertebrates are represented in the *C. elegans *genome, typically with one or at most two members. While redundancy is most likely to be found among members of the same subfamily, members of different subfamilies have also been shown to overlap in function [28]. Not found in *C. elegans *(but in *Drosophila *and vertebrates) is DsCAM, which is thought to play an important role in neuronal wiring by defining the molecular identity of neurons (for a recent review, see [29]). Also not present in the *C. elegans *genome are SynCAMs, a group of four adhesion molecules with three Ig domains that can engage in homo- and heterophilic interactions and can promote synapse development [30], and nectins, which mediate cell adhesion in a number of different contexts [31]. The *C. elegans *genome does contain a single gene with the same domain organization as nectins or SynCAMs (three Ig domains), but at the amino acid level this protein shows no pronounced similarity to either group. This is true for all five IgCAMs in Figure 1, which are listed as not having homologues, illustrating that IgCAMs have continued to evolve after the major metazoan split.

### IgCAM expression

Most of the IgCAMs analysed here have a neuronal expression component. This is similar to previously studied IgCAMs like *unc-5*, *unc-40*, *sax-3 *and *sax-7/lad-1*, all of which are widely expressed in neurons as well [13,16,17,20]. All IgCAMs studied here, with the exception of *rig-3*, are expressed in non-neuronal tissues as well. Overall, every major tissue in *C. elegans *expresses one or more of the IgCAMs. The early onset of expression during embryogenesis suggests that these IgCAMs might have important roles in development. Expression is maintained into adulthood, which points to a continuous requirement for these genes throughout the life cycle. The use of transcriptional GFP reporters does not allow any conclusions about the subcellular localization of the native proteins, which could be an important indicator of their function. Furthermore, we have no guarantee that our expression constructs contain all control elements, leaving the possibility that the observed patterns could be incomplete.

The neuronal expression was characterized in more detail at the cellular level. Expression data for some of the genes studied here has been published previously [12,22-24], but not to this extent. Our analysis revealed that IgCAMs are expressed in a large number of neurons, but with an interesting bias towards the output side of the nervous system. Co-expression of up to five different IgCAMs was mainly observed in classes of neurons that occupy a central position in particular neuronal circuits, most notably command interneurons of the motor circuit and motoneurons themselves. Several of the neurons expressing multiple IgCAMs (AIB, AVA, RMD, SMD) are neurons integrating multiple inputs, which form synaptic connections with many different neurons. It is conceivable that IgCAMs provide part of a recognition system allowing those neurons to identify the correct synaptic partners. Since these IgCAMs are expressed throughout the life cycle of the animal, it is also possible that they are important for maintenance or dynamic changes in synaptic connections. There is evidence for a role of IgCAMs in establishment of synapses as well as synaptic remodelling (for reviews, see [10,32-34]). The IgCAM member SYG-1 in *C. elegans *was shown to localize to synapses in the HSN neuron [22] and is implicated in specifying the position of the synapse in combination with SYG-2, another IgCAM member [23].

### The role of IgCAMs in neuronal development

Several IgCAMs in *C. elegans *have been identified in genetic screens for mutants with neuronal defects. Among those are UNC-5 and UNC-40, the two UNC-6/netrin receptors [13,16] and SAX-3, the receptor for slit [17]. The corresponding mutants have obvious developmental defects leading to uncoordinated movement or even incompletely penetrant embryonic lethality in the case of *sax-3*. More subtle neuronal defects were found in *syg-1 *and *syg-2 *mutants, which affect the placement of a synapse made by the HSN neurons with no further phenotypic consequences [22,23]. The L1 homologue *lad-1/sax-7 *was identified in genetic screens [21] and also the target of a reverse genetic screen [20]. Similarly, mutations were introduced in *wrk-1 *[24], overlapping with our own work, and *lad-2 *[25]. We have extended this reverse genetic approach to most of the remaining members of the family. A common feature of the mutations analyzed here is the absence of a pronounced visible phenotype. Many of the IgCAMs are expressed in various non-neuronal tissues, yet these tissues show no gross defects in any of the single mutants. If these genes were involved in important signalling or adhesion events during embryogenesis, embryonic lethality or at least noticeable morphogenetic defects in some tissues should be the consequence. Such defects are apparent in mutants of other adhesion molecules, such as the classical cadherin *hmr-1 *[35], the fat-like cadherin *cdh-4 *[36] as well as integrins [37]. Within the nervous system we see a similar lack of severe phenotypes in many single mutants. A notable exception is *wrk-1*, which we found to be required for proper navigation of axons in the ventral cord, confirming results of a previous study [24]. Major aspects of neuronal development like cell fate specification or neuronal migration are all unaffected in the neurons analysed. Overall, this is reminiscent of targeted mutations introduced in various IgCAMs in vertebrates. Null alleles of mouse NCAM [2], L1 [38], CHL1 [5], NrCAM [39], TAG-1 [40], NB-3 [41], BIG-2 [42] and BEN/ACLAM/SC1 [43] all show no gross anatomical defects and frequently only limited neurological defects. Only few knock-outs of IgCAMs in mouse are lethal in early postnatal stages. Contactin mutant mice become ataxic after postnatal day 10 and die around postnatal day 18 with a smaller cerebellum [44]. It is interesting to note that orthologs of genes showing severe neuronal defects in *C. elegans *(*unc-5 *and *unc-40*) also show severe defects in the mouse (DCC [45], UNC-5 [46]).

### Genetic interactions among IgCAMs

The lack of a phenotype in single mutants of members of a larger family is typically attributed to functional overlap. This is based on the assumption that closely related family members could functionally replace (at least in part) the mutated gene. The obvious test for such redundancy is to generate multiple mutants of all genes suspected to be functionally related. Within the IgCAM superfamily, which is characterized by widespread biochemical interactions among members of the same as well different subfamilies, it is difficult to predict functionally redundant members purely based on structural features and subfamily. Since IgCAMs most likely act cell-autonomously, we used expression patterns to group genes for genetic interaction studies. We found two cases of genetic interactions: the *rig-1*, -*3*, -*5*, -*6 *quadruple mutant affected command interneuron navigation in the ventral cord and *syg-1*, *wrk-1 *and *ncam-1 *affected directional outgrowth of DA2/DB3 motoneuron commissures. In the second case the three genes involved are co-expressed in the affected neurons, arguing for a simple non-autonomous action of these genes. However, the various classes of command interneurons typically only co-express two of the four genes contributing to the phenotype, so that the observed defects cannot be explained by a simple functional overlap and cell-autonomous action (assuming we have the complete expression pattern of the gene). The marker used to label these neurons (*glr-1*::GFP) is expressed in a total of 11 neurons with axons in the ventral cord. The axons of all these neurons are normally tightly bundled, and it is conceivable that a loss of several adhesion molecules from various neurons in the group could lead to overall weakened adhesion and axons straying from the bundle. The observation that the triple mutant combinations show no enhanced defects compared to single mutants would argue for a threshold effect rather than a gradual loss of adhesion leading to gradually stronger defects.

No further genetic interactions for a range of phenotypes were found in a strain combining all eight mutations. The octuple mutant is viable and fertile and shows no further enhancement of the defects or novel phenotypes compared to either single or multiple mutants analyzed before. This suggests that we did not miss any other interactions with respect to the phenotypes analysed here. A functional overlap among homologs of some of the genes studied here has been documented in other animals [28,47]. The lack of any more severe phenotypes in the octuple mutant is in striking contrast to the pronounced phenotypes found even in single mutants of various other members of the family, most notably *unc-5*, *unc-40 *[48] and *sax-3 *[17]. There are a number of possible reasons for the absence of pronounced phenotypes. First of all, without the tools to detect the native proteins (antibodies) we have currently no conclusive proof that all our alleles are complete loss-of-function alleles. Almost all of them are predicted to truncate the protein before the transmembrane domain and, therefore, would be able to produce a partial protein (most likely secreted). While such a protein fragment is not expected to have residual function, we cannot completely exclude the possibility. Secondly, only a limited set of potential phenotypes was scored, which leaves the possibility that there are phenotypes both in single and multiple mutants still to be discovered – either in cells not analyzed here or for aspects of neuronal development not addressed here. We have not yet analyzed males for any phenotypes and would have missed a role of any of these IgCAMs in male development. Since we did not notice any problems with mating behaviour of males in either single or multiple mutants, those genes located on the X chromosome (of which males have only one copy) collectively do not seem to play a major role in anatomical structures required for mating like the copulatory organs themselves and the nervous system controlling mating behaviour.

Synapse formation is an important aspect within the nervous system, which deserves further attention in the future. There is certainly precedence for IgCAMs being involved in this process in *C. elegans *[22,23] as well as other animals [10,32-34]. However, the overall behaviour of the octuple mutant animals, which seems not severely disturbed, already suggests that connectivity defects (if present) are probably limited as well. One simple final possibility is that most of these genes have no important role during development, and for the most part they are not functionally redundant. However, the striking conservation of this family of proteins across animal phyla down to the exact domain composition suggest a strong evolutionary pressure, which should be based on functional importance.

## Conclusion

Expression analysis of 12 IgCAMs revealed that the majority is expressed in a subset of neurons, suggesting a role in neuronal development and/or function. Analysis of the neuronal expression at the single cell level revealed several hubs of expression in central neurons of the motorcircuit, pointing to potential sites of action. Analysis of eight single mutants revealed limited neuronal defects in certain subsets of neurons, most notably axon navigation defects, confirming that some of these genes indeed have a role during nervous system development. Analysis of multiple mutants revealed two cases of genetic interactions involving three and four IgCAMs, respectively, demonstrating functional overlap among certain family members.

## Materials and methods

### Generation of GFP expression constructs

PCR was used to amplify putative promoter regions from IgCAM genes (primers used are listed in Additional file 4). Resulting PCR products were either fused to the GFP part of a *C. elegans *GFP cloning vector in a second 'fusion PCR' reaction [49] or cloned into the GFP vector pPD95.75 (Fire vector kit, Addgene, Cambridge, MA, USA). Transgenic animals were made by injecting the corresponding purified DNA together with a selectable marker [50]. Two or three independent lines were made for each construct. Since different lines made with the same construct showed the same expression pattern, one was selected for detailed analysis and cell identification. Based on the presence of a significant neuronal expression component, we named previously unnamed IgCAMs either 'rig' for 'neuRonal IG' or 'igcm' for 'IG CaM'. The list of primers used for expression analysis can be found in Additional file 4.

### Isolation of deletion alleles and generation of strain with multiple mutants

Deletion alleles were isolated from a library of animals mutagenized with ethyl methane sulfonate (EMS) using a poison primer approach to identify small deletions in certain region of the gene [51]. PCR primer sets used for deletion detection are listed in Additional file 5; they were designed using AcePrimer [52]. Deleted regions are listed in Additional file 2, and details of the deletion (size, consequences for the protein) are given in Additional file 3. A graphical display of the location of the deletions and the promoter regions used for expression analysis can be found in Figure 2. Strains carrying multiple mutants were generated by crossing the single mutants. Progeny carrying the desired mutant combination were identified by PCR assay. The deletion in *rig-3(hd51) *extends into and potentially affects the neighbouring gene C53B7.7 (Additional file 3). Since *rig-3(hd51) *mutants show interneuron axon guidance defects in combination with *rig-5*, *rig-6 *and *rig-1 *(Table 4), we determined whether C53B7.7 rather than *rig-3 *is involved by rescue experiments with two fosmids: one containing intact copies of both *rig-3 *and C53B7.7 (fosmid WRM0634bC05) and one containing only C53B7.7 (fosmid WRM062bA01). None out of eight lines containing fosmid WRM062bA01 rescued the axonal defects, whereas all four lines containing fosmid WRM0634bC05 did, confirming that the defects observed are due to the deletion in *rig-3*.

### Phenotypic characterisation

The following integrated GFP reporter constructs were used for analysis of axonal defects: *evIs111 [F25B3.3::GFP]*, *rhIs4 [glr-1::GFP; dpy-20(+)]*, *hdIs22 [unc-129::CFP*, *unc-47::DsRed2]*, *hdIs26 [odr-2::CFP*, *sra-6::DsRed2]*. GFP markers were crossed into the deletion mutants and used to score developmental defects in the nervous system, specifically the placement of neuronal cell bodies and the axon trajectories. For phenotypic analysis animals were incubated with 10 mM NaN_3 _in M9 buffer [53] for 1 hour and mounted on agar pads. All strains were cultured at 20°C using standard methods [53]. To evaluate the development and integrity of non-neuronal tissues, adult animals were examined using Nomarski optics and polarized light microscopy (to evaluate muscle structure).

### Microscopy and neuron identification

To identify neurons in GFP reporter strains, the position of cell bodies and axon trajectories were used as diagnostic criteria [26,53,54]. To generate landmarks, markers with known expression (like *rhIs16 [glr-1::CFP; dpy-20(+)]*) were crossed into the IgCAM expression strain. In addition, certain sensory neurons were labelled with DiI [55], effectively generating triple-labelled animals. Stacks of confocal images with 0.2–0.5 μm between focal planes were recorded with a Quorum WaveFX spinning disc system mounted on a Zeiss Axioplan II microscope. Image acquisition and analysis was done with the Volocity software package (Improvision, Coventry, England). Images were inspected manually and cells were identified by overlap or proximity to labelled *glr-1 *expressing interneurons or DiI labelled sensory neurons. Pharyngeal neurons were identified by position and axon trajectory without additional marker. Maximum intensity projections of all focal planes were used to generate images for the figures.

## Abbreviations

FnIII: fibronectin III; GFP: green fluorescent protein; IgCAM: immunoglobulin superfamily cell adhesion molecule; NCAM: neural cell adhesion molecule.

## Competing interests

The authors declare that they have no competing interests.

## Authors' contributions

VS isolated and characterized the single mutants and analyzed the expression patterns. SV-I contributed to the expression analysis. JP contributed to expression analysis and multiple mutant analysis. HH made and analyzed the multiple mutants, characterized the neuronal expression at the single cell level and wrote the manuscript.

## Supplementary Material

Additional file 1 **IgCAM expression in individual neurons**. IgCAM expression in individual neurons. Click here for file

Additional file 2 **Deleted regions in IgCAM mutants**. Deleted regions in IgCAM mutants. Click here for file

Additional file 3 **Description of IgCAM deletion alleles**. Description of IgCAM deletion alleles. Click here for file

Additional file 4 **Primer sequences used to amplify IgCAM promoter regions**. Primer sequences used to amplify IgCAM promoter regions. Click here for file

Additional file 5 **Primer sequences used to isolate deletions**. Primer sequences used to isolate deletions. Click here for file
