# Supplementary material for: IgCAMs redundantly control axon navigation in Caenorhabditis elegans
Source: Neural Dev. 2009 Apr 2;4:13. doi: 10.1186/1749-8104-4-13 (PMC2672934; doi:10.1186/1749-8104-4-13)
Supplement: Additional file 1 — IgCAM expression in individual neurons. IgCAM expression in individual neurons. [file 1749-8104-4-13-S1.doc]

Additional file 1: IgCAM expression in individual neurons

| **Neuron** | **no** | **loc.** | **type** | rig-1 | rig-3 | rig-4 | rig-5 | rig-6 | ncam-1 | wrk-1 | syg-1 | syg-2 | total |
| --- | --- | --- | --- | --- | --- | --- | --- | --- | --- | --- | --- | --- | --- |
| ADE | 2 | head | sensory |  |  |  |  |  |  |  |  |  |  |
| ADF | 2 | head | sensory |  |  |  |  |  |  |  | x |  | 1 |
| ADL | 2 | head | sensory |  |  |  |  |  |  |  | x |  | 1 |
| AFD | 2 | head | sensory |  |  |  |  |  |  |  |  |  |  |
| AQR | 1 | head | sensory |  |  |  |  |  |  |  |  |  |  |
| ASE | 2 | head | sensory |  |  |  |  |  |  |  |  |  |  |
| ASG | 2 | head | sensory |  |  |  |  |  |  |  |  |  |  |
| ASH | 2 | head | sensory |  |  |  |  |  |  |  |  |  |  |
| ASI | 2 | head | sensory |  |  |  |  |  | x | x |  |  | 2 |
| ASJ | 2 | head | sensory |  |  |  |  |  | x | x |  |  | 2 |
| ASK | 2 | head | sensory |  |  |  |  |  |  | x |  |  | 1 |
| AWA | 2 | head | sensory |  |  |  |  |  |  |  |  |  |  |
| AWB | 2 | head | sensory |  |  |  |  |  |  | x |  |  | 1 |
| AWC | 2 | head | sensory |  |  |  |  |  |  |  |  |  |  |
| BAG | 2 | head | sensory |  |  |  |  |  |  |  |  |  |  |
| CEP | 4 | head | sensory |  |  |  |  |  |  |  |  |  |  |
| FLP | 2 | head | sensory |  |  |  |  |  |  |  |  |  |  |
| IL1 | 6 | head | sensory |  |  |  |  |  |  |  |  |  |  |
| IL2 | 6 | head | sensory |  |  |  |  |  |  |  |  |  |  |
| OLL | 2 | head | sensory |  |  |  |  |  |  |  |  |  |  |
| OLQ | 4 | head | sensory |  |  |  |  |  |  |  |  |  |  |
| ADA | 2 | head | inter |  |  |  |  |  |  |  |  |  |  |
| AIA | 2 | head | inter |  |  | x? |  |  |  |  |  |  | 1 |
| AIB | 2 | head | inter | x |  |  |  | x | x | x |  |  | 4 |
| AIM | 2 | head | inter |  |  |  |  |  |  |  |  |  |  |
| AIN | 2 | head | inter | x |  |  | x? |  | x | x? | x |  | 5 |
| AIY | 2 | head | inter |  |  |  |  |  |  | x |  |  |  |
| AIZ | 2 | head | inter |  |  |  |  |  |  |  |  | x? | 1 |
| ALA | 1 | head | inter |  |  |  |  |  |  |  |  |  |  |
| AUA | 2 | head | inter | x |  |  | x? | x |  | x |  |  | 4 |
| AVA | 2 | head | inter |  | x |  |  | x |  | x |  |  | 3 |
| AVB | 2 | head | inter | x |  |  |  | x | x |  |  |  | 3 |
| AVD | 2 | head | inter | x |  |  | x |  |  |  |  |  | 2 |
| AVE | 2 | head | inter |  |  |  |  | x | x | x |  |  | 3 |
| AVF | 2 | head | inter |  |  |  |  |  |  |  |  |  |  |
| AVG | 1 | head | inter |  |  |  |  |  |  |  |  |  |  |
| AVH | 2 | head | inter | x? |  |  |  |  |  |  | x? |  | 2 |
| AVJ | 2 | head | inter | x |  |  |  |  |  |  |  |  | 1 |
| AVK | 2 | head | inter |  |  |  |  |  |  |  |  |  |  |
| RIA | 2 | head | inter |  |  | x? |  |  |  |  |  |  | 1 |
| RIB | 2 | head | inter |  |  |  |  | x |  |  |  |  | 1 |
| RIC | 2 | head | inter | x |  |  | x? | x |  | x |  | x? | 5 |
| RIH | 1 | head | inter |  |  | x? |  |  |  |  |  |  | 1 |
| RIP | 2 | head | inter |  |  |  |  |  |  |  |  |  |  |
| RIR | 1 | head | inter |  |  |  |  |  |  |  |  |  |  |
| RIS | 1 | head | inter |  |  |  |  |  |  |  | x |  | 1 |
| SAA | 4 | head | inter |  |  |  |  | x |  |  | x? |  | 2 |
| SAB | 3 | head | inter |  |  |  |  |  |  |  |  |  |  |
| SIA | 4 | head | inter |  |  |  |  | x |  |  | x |  | 2 |
| SIB | 4 | head | inter |  |  |  |  | x |  |  | x |  | 2 |
| URB | 2 | head | inter |  |  |  |  |  |  |  |  |  |  |
| URX | 2 | head | inter |  |  | x |  |  |  |  |  |  | 1 |
| URY | 4 | head | inter |  |  |  |  |  |  |  |  |  |  |
| AVL | 1 | head | motor |  |  |  |  |  |  |  |  |  |  |
| RID | 1 | head | motor |  |  |  |  |  |  |  |  |  |  |
| RIM | 2 | head | motor |  |  |  |  | x |  |  | x | x? | 3 |
| RIV | 2 | head | motor |  |  |  |  |  |  |  |  |  |  |
| RMD | 6 | head | motor | x |  |  | x | x |  | x |  |  | 4 |
| RME | 4 | head | motor |  |  | x |  | x |  | x |  |  | 3 |
| RMF | 2 | head | motor |  |  |  |  |  |  |  |  |  |  |
| RMG | 2 | head | motor |  |  |  |  |  |  |  |  |  |  |
| RMH | 2 | head | motor |  |  |  |  |  |  |  |  |  |  |
| SMB | 4 | head | motor |  |  |  |  |  |  |  |  |  |  |
| SMD | 4 | head | motor | x |  |  | x | x |  | x |  |  | 4 |
| URA | 4 | head | motor |  |  |  |  |  |  | x? |  |  | 1 |
| ALM | 2 | body | sensory |  |  |  |  |  |  |  |  |  |  |
| AVM | 1 | body | sensory |  |  |  |  |  |  |  |  |  |  |
| PDE | 2 | body | sensory |  |  |  |  |  |  |  |  |  |  |
| BDU | 2 | body | inter |  |  |  |  |  |  |  |  |  |  |
| CAN | 2 | body | inter |  |  |  |  |  |  |  |  |  |  |
| SDQ | 2 | body | inter |  |  |  |  |  |  |  |  |  |  |
| HSN | 2 | body | motor |  |  |  |  |  |  |  |  |  |  |
| ALN | 2 | tail | inter |  |  |  |  |  |  |  |  | x | 1 |
| DVA | 1 | tail | inter |  |  |  |  |  |  | x? |  |  | 1 |
| DVC | 1 | tail | inter |  |  |  |  |  |  |  |  |  |  |
| LUA | 2 | tail | inter |  |  |  |  |  |  |  |  |  |  |
| PLN | 2 | tail | inter |  |  |  |  |  |  |  |  | x | 1 |
| PVC | 2 | tail | inter | x |  |  |  | x | x |  |  |  | 3 |
| PVD | 2 | tail | inter |  |  |  |  |  |  |  |  |  |  |
| PVP | 2 | tail | inter |  |  |  |  |  |  |  |  |  |  |
| PVQ | 2 | tail | inter |  |  |  |  |  |  |  |  |  |  |
| PVR | 1 | tail | inter |  |  |  |  |  |  |  |  |  |  |
| PVW | 2 | tail | inter |  |  |  |  |  |  |  |  |  |  |
| DVB | 1 | tail | motor |  |  |  |  |  |  |  |  |  |  |
| PDA | 1 | tail | motor |  |  |  |  |  |  | x |  |  | 1 |
| PDB | 1 | tail | motor |  |  |  |  |  |  |  |  |  |  |
| PVN | 2 | tail | motor |  |  |  |  |  |  |  |  |  |  |
| PHA | 2 | tail | sensory |  |  |  |  |  |  |  |  |  |  |
| PHB | 2 | tail | sensory |  |  |  |  |  |  |  |  |  |  |
| PHC | 2 | tail | sensory |  |  |  |  |  |  |  |  |  |  |
| PLM | 2 | tail | sensory |  |  |  |  |  |  | x? |  |  | 1 |
| PQR | 1 | tail | sensory |  |  |  |  |  |  |  |  |  |  |
| PVM | 1 | tail | sensory |  |  |  |  |  |  |  |  |  |  |
| PVT | 1 | vnc | inter |  |  |  |  |  |  |  |  | x | 1 |
| RIF | 2 | vnc | inter |  |  |  | x? | x |  |  |  |  | 2 |
| RIG | 2 | vnc | inter |  |  |  |  |  |  | x | x |  | 2 |
| AS | 11 | vnc | motor |  |  |  |  |  |  |  |  |  |  |
| DA | 9 | vnc | motor |  |  | x |  | x | x | x | x |  | 5 |
| DB | 7 | vnc | motor |  |  |  |  | x | x | x | x |  | 4 |
| DD | 6 | vnc | motor |  |  |  |  |  |  | x | x |  | 2 |
| VA | 12 | vnc | motor |  |  |  |  | x |  |  | x |  | 2 |
| VB | 11 | vnc | motor |  |  |  |  | x |  |  | x |  | 2 |
| VC | 6 | vnc | motor |  |  |  |  |  |  |  |  |  |  |
| VD | 13 | vnc | motor |  |  |  |  |  |  |  |  |  |  |
| I1 | 2 | pha | pha_inter |  | x |  |  |  |  |  |  |  | 1 |
| I2 | 2 | pha | pha_inter | x |  |  | x |  |  |  |  |  | 2 |
| I3 | 1 | pha | pha_inter |  |  |  |  | x |  |  |  |  | 1 |
| I4 | 1 | pha | pha_inter |  | x |  |  |  |  |  |  |  | 1 |
| I5 | 1 | pha | pha_inter |  |  |  |  |  |  |  |  |  |  |
| I6 | 1 | pha | pha_inter |  |  |  |  |  |  |  |  |  |  |
| MC | 2 | pha | pha_inter |  |  |  | x | x |  |  |  |  | 2 |
| M1 | 1 | pha | pha_mot |  |  |  |  | x? |  |  |  |  | 1 |
| M2 | 2 | pha | pha_mot |  |  |  | x? |  | x |  |  |  | 2 |
| M3 | 2 | pha | pha_mot |  |  |  | x? |  |  |  | x? |  | 2 |
| M4 | 1 | pha | pha_mot |  | x |  | x |  |  |  | x |  | 3 |
| M5 | 1 | pha | pha_mot |  |  |  |  | x |  |  |  |  | 1 |
| MI | 1 | pha | pha_mot |  |  |  |  |  |  |  |  |  |  |
| NSM | 2 | pha | pha_mot |  | x |  |  | x | x |  |  |  | 3 |
|  |  |  |  | 12 | 5 | 6 | 12 | 25 | 11 | 22 | 17 | 6 | total |

head: cell body in head ganglia

body: cell body along the body

tail: cell body in tail ganglia

vnc: cell body in ventral nerve cord

pha: pharyngeal neuron

sensory: sensory neuron

inter: interneuron

motor: motor neuron

pha_inter: pharyngeal interneuron

pha_motor pharyngeal motor neuron

x: confident

x?: not entirely sure
