# Supplementary material for: IgCAMs redundantly control axon navigation in Caenorhabditis elegans
Source: Neural Dev. 2009 Apr 2;4:13. doi: 10.1186/1749-8104-4-13 (PMC2672934; doi:10.1186/1749-8104-4-13)
Supplement: Additional file 2 — Deleted regions in IgCAM mutants. Deleted regions in IgCAM mutants. [file 1749-8104-4-13-S2.doc]

Additional file 2: Deleted regions in IgCAM mutants

| **allele** | **chr.** | **deleted region** | **flanking sequences** |
| --- | --- | --- | --- |
| *rig-4(hd47)* | IV | 7992138-7992880 | gcatcattccaattaaaaggaacat--taaaaaaaataaagcaagatctatt |
| *rig-1(hd15)* | X | 8661473-8662000 | gaagctcgcatgcaatcagttgtat--tcgtagttttttataaaaaactttt |
| *ncam-1(hd49)* | X | 700866-701564 | ctacccgaactatggaaaatgttca--aatcaagtgcagctgacaaatatca |
| *wrk-1(hd45)* | X | 8390873-8391141 | tggttcattatacccctaatctctt--acatgttgctggtaagcattttcaa |
| *rig-3(hd51)* | X | 6859601-6861122 | agtttttttttgaatgttaatgttc--gacttcatcattcttgagtcacttg |
| *syg-1(hd18)* | X | 2514383-2515391 | ttctcaatattgcgtgtctctcttt--atattttaggcctgagttttattgc |
| *rig-5(hd48)* | I | 9574483-9575411 | atgggatgggaaaaggatggagaac--caaaagaatggtcgctttttcgttt |
| *rig-6(gk376)* | II | 4805822-4804935 | agttttccttttcgcattggt--atttcttgtgggcaccgataa |
