# Supplementary material for: IgCAMs redundantly control axon navigation in Caenorhabditis elegans
Source: Neural Dev. 2009 Apr 2;4:13. doi: 10.1186/1749-8104-4-13 (PMC2672934; doi:10.1186/1749-8104-4-13)
Supplement: Additional file 3 — Description of IgCAM deletion alleles. Description of IgCAM deletion alleles. [file 1749-8104-4-13-S3.doc]

Additional file 3: Description of IgCAM deletion alleles

| **Allele** | **deletion size** | **description** |
| --- | --- | --- |
| *rig-4(hd47)* | 742 bp | frame shift after amino acid 2098; deletes most of the intracellular domain |
| *rig-1(hd15)* | 527 bp | frame shift after amino acid 79 |
| *ncam-1(hd49)* | 698 bp | frame shift after amino acid 600 (in the first FnIII domain and before the transmembrane domain) |
| *wrk-1(hd45)* | 268 bp | frameshift after amino acid 155 |
| *rig-3(hd51)* | 1521 bp | deletes C-terminal part after amino acid 346 (transmembrane domain and entire intracellular domain); also deletes part of the last exon of the neighbouring gene C53B7.7, a putative metallopeptidase with no clear ortholog in other species |
| *syg-1(hd18)* | 1008 bp | deletes first exon |
| *rig-5(hd48)* | 928 bp | frame shift and stop codon after amino acid 199 (truncates the protein at end of the first immunoglobulin domain) |
| *rig-6(gk376)* | 888 bp | deletes first exon of *rig-6a*; other splice variants not affected |
