# Supplementary material for: IgCAMs redundantly control axon navigation in Caenorhabditis elegans
Source: Neural Dev. 2009 Apr 2;4:13. doi: 10.1186/1749-8104-4-13 (PMC2672934; doi:10.1186/1749-8104-4-13)
Supplement: Additional file 4 — Primer sequences used to amplify IgCAM promoter regions. Primer sequences used to amplify IgCAM promoter regions. [file 1749-8104-4-13-S4.doc]

Additional file 4: Primer sequences used to amplify IgCAM promoter regions

| **gene** | **name** | **Sequence** |
| --- | --- | --- |
| *rig-1* | sv_K09E2.4_1 | gggacgaagtaggaataatgtgga |
|  | sv_K09E2.4_2 | gcaatcattgtagaattcctcatt |
| *rig-3* | VS_C53B7.1_1 | ggaaaaatgtgagatcttcg |
|  | VS_C53B7.1_2 | gaatgaagttcttctgcaagg |
| *rig-4* | VS_Y42H9B.2_1 | ttcatttgtgtccgtccgttgc |
|  | HH_Y42H9B.2_2 | cgttcctccatgtgtacttctcct |
| *rig-5* | VS_rig-5fu_1 | tcctaatccgtttgctttccc |
|  | VS_rig-5fu_2 | gcaaacttgtgatgtttctgg |
|  | VS_rig-5fu_3 | ccgagatagtgcgaagcaagt |
| *rig-6a* | VS_C33F10.5afu_1 | aaagagataagggtagtcgct |
|  | VS_C33F10.5afu_2 | tggaaacctatgtagtagctg |
|  | VS_C33F10.5afu_3 | ccaaaaagaagatgtgccgac |
| *rig-6c* | VS_C33F10.5cfu_1 | ccatctcaaaccgcttcagca |
|  | VS_C33F10.5cfu_2 | cacacttgtactttccagcat |
|  | VS_C33F10.5cfu_3 | cctaactccctacgcttttcc |
| *ncam-1a/c* | VS_F02G3.1_1 | atctcgtgagccgtgttgttga |
| *ncam-1a* | VS_F02G3.1afu_2 | tcatcctgtgctttgccggtg |
| *ncam-1c* | VS_F02G3.1cfu_2 | tgacacatttgcgccgagttg |
| *syg-1* | K02E10.8_aag_1 | taatggtcagcccgatagaaatag |
|  | K02E10.8_aag_2 | ctgccaccgtaccTtctttctgcc |
| *syg-2* | Ce_neph_pr1 | atcttgtgcctcccatctaaa |
|  | Ce_neph_pr2 | tggaatcactccacgtctgaa |
| *wrk-1* | sv_F41D9.3_1 | gtctcaatactcaccacccttt |
|  | sv_F41D9.3_2 | agcagaaccaatttcatcatgc |
| *igcm-1* | VS_F39H12.4_1 | atgagatcttaccgacttccttatgg |
|  | VS_F39H12.4fu_2 | ccctcccGtctggttggtagaatt |
|  | VS_F39H12.4fu_3 | caccctttccgcatgtggttt |
| *igcm-2* | VS_SSSD1.1_1 | catttctcgggaacgccaagat |
|  | VS_SSSD1.1fu_2 | TTagatttgagactgcttgtattct |
|  | VS_SSSD1.1fu_3 | gaatacacagacgatggtagt |
| *igcm-3* | T02C5.3_pr1 | agcctctccttccaacacattcag |
|  | HH_T02C5.3_pr2 | ctacaaaatgcttgtaataaagtcga |
| *GFP* | HH_GFPfu_2 | aagggcccgtacggccgactagtagg |
|  | HH_GFPfu_3 | agcttgcatgcctgcaggtcgact |
|  | HH_GFPfu_4 | ggaaacagttatgtttggtatattggg |

Odd numbers designate upstream primers, even numbers downstream primers
