# Supplementary material for: IgCAMs redundantly control axon navigation in Caenorhabditis elegans
Source: Neural Dev. 2009 Apr 2;4:13. doi: 10.1186/1749-8104-4-13 (PMC2672934; doi:10.1186/1749-8104-4-13)
Supplement: Additional file 5 — Primer sequences used to isolate deletions. Primer sequences used to isolate deletions. [file 1749-8104-4-13-S5.doc]

Additional file 5: Primer sequences used to isolate deletions

| **allele** | **primer name** | **sequence** |
| --- | --- | --- |
| *rig-4(hd47)* | HH_Y42H9B.2_dx1 | ttctggccaagttggatagttt |
|  | HH_Y42H9B.2_dx2 | gaaaacggcaagaattttgaac |
|  | HH_Y42H9B.2_dp1 | atattggcagctgatgttcctt |
|  | HH_Y42H9B.2_di1 | atgcttggcctaaagaagacag |
|  | HH_Y42H9B.2_di2 | ccttcactcgtgctatcagttg |
| *rig-1(hd15)* | HH_K09E2.4_dx1 | gccaccccactcacttttta |
|  | HH_K09E2.4_dx2 | ttgcacatccctcattgtgt |
|  | HH_K09E2.4_di1 | agcgttaagtttgacggctg |
|  | HH_K09E2.4_di2 | tttgatctgcccatgtttga |
|  | HH_K09E2.4_dp1 | aagctcgcatgcaatcagtt |
| *ncam-1(hd49)* | HH_F02G3.1_dx1 | acctcagaaaattccgaagaaac |
|  | HH_F02G3.1_dx2 | ggactaaaaataccacctgaccc |
|  | HH_F02G3.1_di1 | tgaacgatgaggaagaccatact |
|  | HH_F02G3.1_di2 | ttccttgtacgtgacagactacg |
|  | HH_F02G3.1_dp2 | tttgaagtcaagtttggaatggt |
| *wrk-1(hd45)* | HH_F41D9.3_dx5 | cacgaccgaattttacaactga |
|  | HH_F41D9.3_dx6 | agatgagtgattgatggcacac |
|  | HH_F41D9.3_di5 | gcacgctgtacagaactgtgtt |
|  | HH_F41D9.3_di6 | ttgcagctttcctcctatcatt |
|  | HH_F41D9.3_dp6 | aagtccaagaatcagcagaacc |
| *rig-3(hd51)* | HH_C53B7.1_dx3 | tttctcaaggagcacatctcaa |
|  | HH_C53B7.1_dx4 | ctgacggtcttccgtatttttc |
|  | HH_C53B7.1_di3 | aacgatttgggcacttacaaat |
|  | HH_C53B7.1_di4 | gtttaaggttcctgatgcttgc |
|  | HH_C53B7.1_dp3 | agatccagatcttccaatgacc |
| *syg-1(hd18)* | HH_K02E10.8_dx1 | tgctttgtcagtgccttttg |
|  | HH_K02E10.8_dx2 | aaaccggaggagattgtgtg |
|  | HH_K02E10.8_di1 | gcacttatctggtggccttc |
|  | HH_K02E10.8_di2 | attccgagatttgacaagcg |
|  | HH_K02E10.8_dp2 | gtctgccaccgtaccatctt |
| *rig-5(hd48)* | HH_C36F7.4a_dx1 | ttttattttggagggtgagaagag |
|  | HH_C36F7.4a_dx2 | ttgttttaaaatagcatgcctgaa |
|  | HH_C36F7.4a_di1 | aattgacaacaacgacgatttaga |
|  | HH_C36F7.4a_di2 | acatacatgcgatatttgagtgct |
|  | HH_C36F7.4a_dp2 | tccaaaatgagatgattgaacatt |

Each primer set consists of en external primer pair (dx), a nested internal primer pair (di) and a ‘poison’ primer (dp).
